# 3,4-Di­methyl­thieno[2,3-*b*]thio­phene-2,5-dicarbo­nitrile

**DOI:** 10.1107/S1600536813017960

**Published:** 2013-07-17

**Authors:** Yahia Nasser Mabkhot, S. S. Al-Showiman, Assem Barakat, M. Iqbal Choudhary, Sammer Yousuf

**Affiliations:** a Department of Chemistry, College of Science, King Saud University, PO Box 2455, Riyadh 11451, Saudi Arabia; b Department of Chemistry, Faculty of Science, Alexandria University, PO Box 426, Ibrahimia- 21321 Alexandria, Egypt; c H.E.J. Research Institute of Chemistry, International Center for Chemical and Biological Sciences, University of Karachi, Karachi 75270, Pakistan

## Abstract

The asymmetric unit of the title compound, C_10_H_6_N_2_S_2_, contains two crystallographically independent but conformationally similar mol­ecules. The fused thio­phene ring cores are almost planar [maximum deviation = 0.027 (3) Å] with the thio­phene rings forming dihedral angles of 0.5 (4)° in one mol­ecule and 1.91 (4)° in the other. The crystal packing is stabilized only by van der Waals inter­actions.

## Related literature

For the biological activity of thio­phene derivatives, see: Mabkhot *et al.* (2013 ▶); Mishra *et al.* (2011 ▶). For the synthesis of fused heterocyclic compounds, see: Cornel & Kirsch (2001 ▶); Mashraqui *et al.* (1999 ▶). For crystal data for related thio­phene compounds, see: Gunasekaran *et al.* (2009 ▶); Mashraqui *et al.* (2004 ▶).
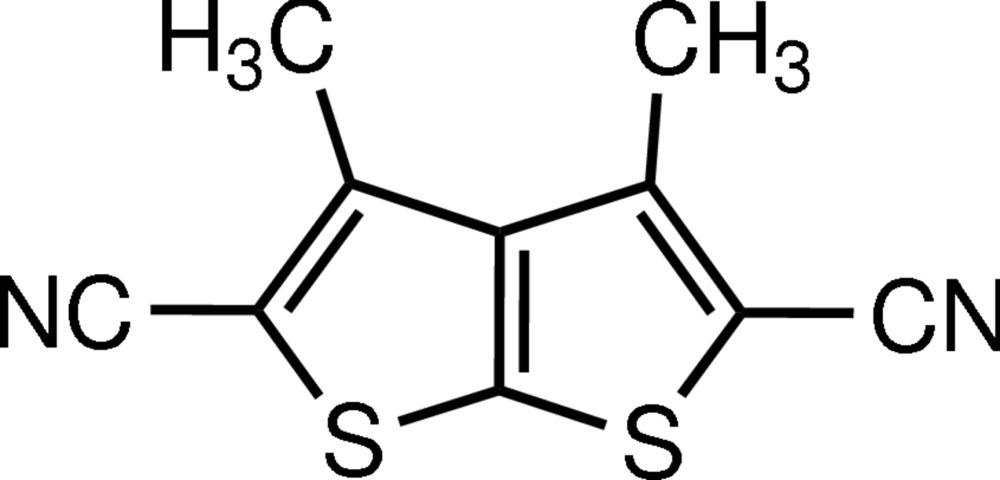


## Experimental

### 

#### Crystal data

C_10_H_6_N_2_S_2_
*M* *_r_* = 218.31
Triclinic, 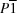

*a* = 7.2573 (11) Å
*b* = 10.1538 (15) Å
*c* = 13.665 (2) Å
α = 94.467 (3)°
β = 99.120 (4)°
γ = 95.850 (4)°
*V* = 984.5 (3) Å^3^
*Z* = 4
Mo *K*α radiation
μ = 0.50 mm^−1^
*T* = 273 K
0.37 × 0.15 × 0.11 mm

#### Data collection

Bruker SMART APEX CCD area-detector diffractometer
Absorption correction: multi-scan (*SADABS*; Bruker, 2000 ▶) *T*_min_ = 0.838, *T*_max_ = 0.947
13821 measured reflections
4912 independent reflections
3074 reflections with *I* > 2σ(*I*)
*R*_int_ = 0.053

#### Refinement

*R*[*F* ^2^ > 2σ(*F* ^2^)] = 0.055
*wR*(*F* ^2^) = 0.132
*S* = 0.99
4912 reflections
257 parameters
H-atom parameters constrained
Δρ_max_ = 0.37 e Å^−3^
Δρ_min_ = −0.24 e Å^−3^

### 

Data collection: *SMART* (Bruker, 2000 ▶); cell refinement: *SAINT* (Bruker, 2000 ▶); data reduction: *SAINT*; program(s) used to solve structure: *SHELXS97* (Sheldrick, 2008 ▶); program(s) used to refine structure: *SHELXL97* (Sheldrick, 2008 ▶); molecular graphics: *SHELXTL* (Sheldrick, 2008 ▶); software used to prepare material for publication: *SHELXTL*, *PARST* (Nardelli, 1995 ▶) and *PLATON* (Spek, 2009 ▶).

## Supplementary Material

Crystal structure: contains datablock(s) global, I. DOI: 10.1107/S1600536813017960/rz5077sup1.cif

Structure factors: contains datablock(s) I. DOI: 10.1107/S1600536813017960/rz5077Isup2.hkl

Supplementary material file. DOI: 10.1107/S1600536813017960/rz5077Isup3.cml

Additional supplementary materials:  crystallographic information; 3D view; checkCIF report

## Figures and Tables

**Fig. 1.**
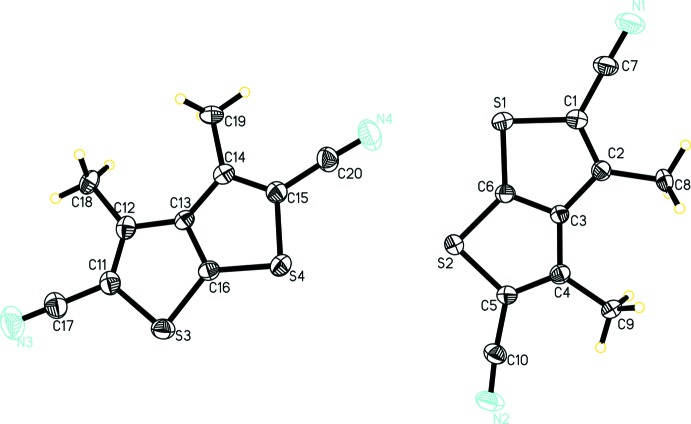
The molecular structure of the title compound with displacement ellipsoids drawn at the 30% probability level.

**Fig. 2.**
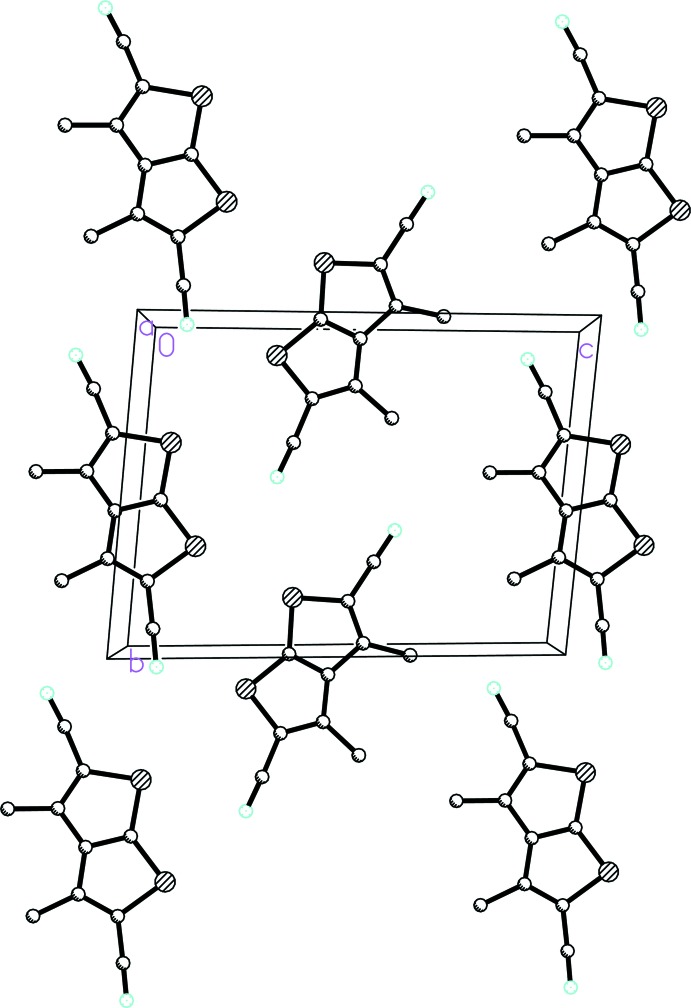
Partial packing diagram of the title compound. Hydrogen atoms are omitted for clarity.

## supplementary crystallographic information

### Comment

Thiophene moieties containing heterocyclic compounds are known to have a number of biological activities, including anti-inflammatory, anti-oxidant and anti-glycation properties *etc*. (Mabkhot *et al.*, 2013; Mishra *et al.* 2011). The title compound is a thiophene derivative, composed of two fused thiophene rings. It was synthesized as part of our ongoing research towards the synthesis of novel chemical entities with diverse biological activities.

The title, compound C_10_H_6_N_2_S_2_, contains two independent (S1–S2/C1–C6 and S3–S4/C11–C16) in the asymmetric unit (Fig. 1). Structurally it is similar to the previous reported compound diethyl 3,4-*bis*(acetoxymethyl)thieno-[2,3-*b*]thiophene-2,5-dicarboxylate (Gunasekaran *et al.*, 2009) with the difference that four acetoxy methyl substituents has been replaced by two nitrile and two methyl substituents. The fused thiophene ring cores are almost planar [maximum deviation 0.027 (3) Å for atom C16] as indicated by the dihedral angles formed by the thiophene rings of 0.5 (4)° in one molecule and 1.91 (4)° in the other. The crystal packing (Fig. 2) is stabilized only by van der Waals interactions.

### Experimental

The title compound was synthesized by following the procedure described in the literature (Mashraqui *et al.*, 1999; Cornel *et al.*, 2001). The compound was crystallized by using a mixture of dimethyl formamide (DMF) and dichloromethane (CH_2_Cl_2_) (1:1 *v*/*v*) at room temperature to obtain light brown crystals. M. p. 498 K. Anal. calcd. for C_10_H_6_N_2_S_2_: C, 55.04; H, 2.75; N, 12.84. Found: C, 54.82; H, 2.92; N, 12.97.

Spectral Data: IR (KBr, cm^-1^): 2964, 2213 cm-1; ^1^H-NMR (400 MHz, CDCl_3_): δ 2.69 (s, 6H, CH_3_); ^13^C-NMR (100 MHz, CDCl_3_): δ 14.8 (2 CH_3_), 108.5 (2 CAr), 113.3 (2 CN), 134.1 (CAr), 143.1 (2 CAr), 150.8 p.p.m. (CAr).

### Refinement

H Atoms were positioned geometrically and refined as riding, with C—H = 0.96 Å and with *U*_iso_(H) = 1.5*U*_eq_(C).

### Crystal data

**Table:**

C_10_H_6_N_2_S_2_	*Z* = 4
*M**_r_* = 218.31	*F*(000) = 448
Triclinic, *P*1	*D*_x_ = 1.473 Mg m^−^^3^
Hall symbol: -P 1	Mo *K*α radiation, λ = 0.71073 Å
*a* = 7.2573 (11) Å	Cell parameters from 1631 reflections
*b* = 10.1538 (15) Å	θ = 1.5–28.4°
*c* = 13.665 (2) Å	µ = 0.50 mm^−^^1^
α = 94.467 (3)°	*T* = 273 K
β = 99.120 (4)°	Block, brown
γ = 95.850 (4)°	0.37 × 0.15 × 0.11 mm
*V* = 984.5 (3) Å^3^	

### Data collection

**Table:**

Bruker SMART APEX CCD area-detector diffractometer	4912 independent reflections
Radiation source: fine-focus sealed tube	3074 reflections with *I* > 2σ(*I*)
Graphite monochromator	*R*_int_ = 0.053
ω scan	θ_max_ = 28.4°, θ_min_ = 1.5°
Absorption correction: multi-scan (*SADABS*; Bruker, 2000)	*h* = −9→9
*T*_min_ = 0.838, *T*_max_ = 0.947	*k* = −13→13
13821 measured reflections	*l* = −18→18

### Refinement

**Table:**

Refinement on *F*^2^	Primary atom site location: structure-invariant direct methods
Least-squares matrix: full	Secondary atom site location: difference Fourier map
*R*[*F*^2^ > 2σ(*F*^2^)] = 0.055	Hydrogen site location: inferred from neighbouring sites
*wR*(*F*^2^) = 0.132	H-atom parameters constrained
*S* = 0.99	*w* = 1/[σ^2^(*F*_o_^2^) + (0.0565*P*)^2^] where *P* = (*F*_o_^2^ + 2*F*_c_^2^)/3
4912 reflections	(Δ/σ)_max_ = 0.001
257 parameters	Δρ_max_ = 0.37 e Å^−^^3^
0 restraints	Δρ_min_ = −0.24 e Å^−^^3^

### Special details

**Table:**

Geometry. All e.s.d.'s (except the e.s.d. in the dihedral angle between two l.s. planes) are estimated using the full covariance matrix. The cell e.s.d.'s are taken into account individually in the estimation of e.s.d.'s in distances, angles and torsion angles; correlations between e.s.d.'s in cell parameters are only used when they are defined by crystal symmetry. An approximate (isotropic) treatment of cell e.s.d.'s is used for estimating e.s.d.'s involving l.s. planes.
Refinement. Refinement of *F*^2^ against ALL reflections. The weighted *R*-factor *wR* and goodness of fit *S* are based on *F*^2^, conventional *R*-factors *R* are based on *F*, with *F* set to zero for negative *F*^2^. The threshold expression of *F*^2^ > σ(*F*^2^) is used only for calculating *R*-factors(gt) *etc*. and is not relevant to the choice of reflections for refinement. *R*-factors based on *F*^2^ are statistically about twice as large as those based on *F*, and *R*- factors based on ALL data will be even larger.

### Fractional atomic coordinates and isotropic or equivalent isotropic displacement parameters (Å^2^)

**Table:**

	*x*	*y*	*z*	*U*_iso_*/*U*_eq_	
S1	0.18703 (11)	0.32325 (7)	0.83846 (5)	0.0460 (2)	
S2	0.24419 (10)	0.62763 (7)	0.91346 (5)	0.0435 (2)	
S3	0.38935 (11)	1.16617 (7)	0.61752 (6)	0.0477 (2)	
S4	0.37946 (11)	0.89010 (8)	0.70279 (5)	0.0473 (2)	
N1	0.1667 (4)	−0.0317 (3)	0.8989 (2)	0.0718 (9)	
N2	0.3550 (4)	0.8848 (3)	1.1405 (2)	0.0706 (9)	
N3	0.2705 (5)	1.3690 (3)	0.4034 (3)	0.0902 (11)	
N4	0.2415 (4)	0.5277 (3)	0.6753 (2)	0.0827 (10)	
C1	0.1990 (4)	0.2211 (3)	0.9361 (2)	0.0424 (7)	
C2	0.2290 (4)	0.2874 (3)	1.0280 (2)	0.0381 (6)	
C3	0.2441 (3)	0.4276 (2)	1.02172 (19)	0.0336 (6)	
C4	0.2755 (4)	0.5432 (3)	1.0910 (2)	0.0384 (6)	
C5	0.2804 (4)	0.6552 (3)	1.0431 (2)	0.0396 (7)	
C6	0.2251 (4)	0.4597 (3)	0.9243 (2)	0.0371 (6)	
C7	0.1799 (4)	0.0807 (3)	0.9144 (2)	0.0520 (8)	
C8	0.2489 (4)	0.2224 (3)	1.1236 (2)	0.0471 (7)	
H8A	0.2582	0.1294	1.1097	0.071*	
H8B	0.1410	0.2332	1.1547	0.071*	
H8C	0.3601	0.2632	1.1674	0.071*	
C9	0.3013 (4)	0.5424 (3)	1.20166 (19)	0.0447 (7)	
H9A	0.3111	0.6320	1.2319	0.067*	
H9B	0.4138	0.5039	1.2246	0.067*	
H9C	0.1954	0.4909	1.2196	0.067*	
C10	0.3195 (4)	0.7857 (3)	1.0941 (2)	0.0474 (7)	
C11	0.2885 (4)	1.1564 (3)	0.4924 (2)	0.0457 (7)	
C12	0.2220 (4)	1.0317 (3)	0.4497 (2)	0.0410 (7)	
C13	0.2546 (3)	0.9366 (3)	0.52151 (18)	0.0341 (6)	
C14	0.2139 (4)	0.7963 (3)	0.5223 (2)	0.0389 (6)	
C15	0.2732 (4)	0.7597 (3)	0.6151 (2)	0.0441 (7)	
C16	0.3434 (4)	0.9965 (3)	0.6131 (2)	0.0378 (6)	
C17	0.2795 (5)	1.2748 (3)	0.4434 (3)	0.0590 (9)	
C18	0.1286 (4)	0.9979 (3)	0.34337 (19)	0.0452 (7)	
H18A	0.1082	1.0783	0.3128	0.068*	
H18B	0.0102	0.9451	0.3412	0.068*	
H18C	0.2077	0.9486	0.3081	0.068*	
C19	0.1235 (4)	0.6996 (3)	0.4357 (2)	0.0453 (7)	
H19A	0.1213	0.6108	0.4554	0.068*	
H19B	0.1938	0.7076	0.3822	0.068*	
H19C	−0.0028	0.7184	0.4140	0.068*	
C20	0.2548 (4)	0.6297 (3)	0.6466 (2)	0.0546 (8)	

### Atomic displacement parameters (Å^2^)

**Table:**

	*U*^11^	*U*^22^	*U*^33^	*U*^12^	*U*^13^	*U*^23^
S1	0.0602 (5)	0.0394 (4)	0.0360 (4)	0.0018 (3)	0.0067 (3)	−0.0035 (3)
S2	0.0552 (5)	0.0359 (4)	0.0397 (4)	0.0042 (3)	0.0079 (3)	0.0063 (3)
S3	0.0538 (5)	0.0395 (4)	0.0488 (5)	0.0045 (3)	0.0102 (4)	−0.0032 (3)
S4	0.0534 (5)	0.0508 (5)	0.0343 (4)	0.0019 (4)	0.0001 (3)	0.0040 (3)
N1	0.100 (2)	0.0396 (16)	0.075 (2)	−0.0008 (16)	0.0267 (18)	−0.0085 (15)
N2	0.096 (2)	0.0385 (16)	0.074 (2)	0.0009 (15)	0.0168 (18)	−0.0122 (15)
N3	0.126 (3)	0.067 (2)	0.096 (3)	0.034 (2)	0.044 (2)	0.036 (2)
N4	0.094 (2)	0.059 (2)	0.083 (2)	−0.0117 (17)	−0.0187 (18)	0.0285 (18)
C1	0.0446 (16)	0.0374 (16)	0.0437 (17)	0.0029 (13)	0.0064 (13)	−0.0007 (13)
C2	0.0362 (15)	0.0378 (15)	0.0390 (16)	0.0041 (12)	0.0023 (12)	0.0039 (13)
C3	0.0340 (14)	0.0327 (14)	0.0331 (15)	0.0031 (11)	0.0038 (11)	0.0025 (12)
C4	0.0351 (15)	0.0411 (16)	0.0375 (16)	0.0034 (12)	0.0030 (12)	0.0016 (13)
C5	0.0426 (16)	0.0355 (15)	0.0387 (16)	0.0024 (12)	0.0038 (13)	−0.0002 (13)
C6	0.0396 (15)	0.0341 (15)	0.0364 (16)	0.0044 (12)	0.0046 (12)	−0.0001 (12)
C7	0.063 (2)	0.0372 (17)	0.055 (2)	0.0003 (15)	0.0145 (16)	−0.0051 (15)
C8	0.0604 (19)	0.0353 (16)	0.0450 (18)	0.0048 (14)	0.0022 (14)	0.0143 (14)
C9	0.0579 (18)	0.0425 (16)	0.0303 (15)	0.0027 (14)	0.0015 (13)	−0.0020 (13)
C10	0.0543 (18)	0.0351 (16)	0.0538 (19)	0.0085 (14)	0.0114 (15)	0.0007 (15)
C11	0.0480 (17)	0.0458 (18)	0.0489 (18)	0.0131 (14)	0.0175 (14)	0.0102 (15)
C12	0.0351 (15)	0.0507 (18)	0.0424 (17)	0.0122 (13)	0.0152 (13)	0.0097 (14)
C13	0.0290 (13)	0.0421 (16)	0.0314 (15)	0.0047 (12)	0.0072 (11)	0.0005 (12)
C14	0.0345 (14)	0.0447 (17)	0.0368 (16)	0.0044 (12)	0.0060 (12)	0.0004 (13)
C15	0.0454 (16)	0.0437 (17)	0.0422 (17)	0.0028 (13)	0.0050 (13)	0.0048 (14)
C16	0.0367 (15)	0.0398 (15)	0.0370 (16)	0.0055 (12)	0.0077 (12)	−0.0003 (12)
C17	0.070 (2)	0.050 (2)	0.067 (2)	0.0178 (17)	0.0297 (19)	0.0122 (18)
C18	0.0427 (16)	0.063 (2)	0.0307 (15)	0.0129 (14)	0.0007 (13)	0.0102 (14)
C19	0.0459 (17)	0.0402 (16)	0.0440 (17)	−0.0025 (13)	0.0009 (13)	−0.0084 (13)
C20	0.057 (2)	0.050 (2)	0.052 (2)	−0.0026 (16)	−0.0051 (15)	0.0129 (16)

### Geometric parameters (Å, º)

**Table:**

S1—C6	1.714 (3)	C8—H8A	0.9600
S1—C1	1.750 (3)	C8—H8B	0.9600
S2—C6	1.716 (3)	C8—H8C	0.9600
S2—C5	1.745 (3)	C9—H9A	0.9600
S3—C16	1.716 (3)	C9—H9B	0.9600
S3—C11	1.741 (3)	C9—H9C	0.9600
S4—C16	1.703 (3)	C11—C12	1.360 (4)
S4—C15	1.742 (3)	C11—C17	1.423 (4)
N1—C7	1.136 (4)	C12—C13	1.439 (3)
N2—C10	1.130 (4)	C12—C18	1.501 (4)
N3—C17	1.140 (4)	C13—C16	1.377 (3)
N4—C20	1.137 (4)	C13—C14	1.427 (4)
C1—C2	1.351 (4)	C14—C15	1.365 (4)
C1—C7	1.421 (4)	C14—C19	1.495 (4)
C2—C3	1.428 (3)	C15—C20	1.420 (4)
C2—C8	1.502 (4)	C18—H18A	0.9600
C3—C6	1.384 (3)	C18—H18B	0.9600
C3—C4	1.425 (4)	C18—H18C	0.9600
C4—C5	1.356 (4)	C19—H19A	0.9600
C4—C9	1.495 (4)	C19—H19B	0.9600
C5—C10	1.428 (4)	C19—H19C	0.9600
			
C6—S1—C1	89.13 (13)	H9B—C9—H9C	109.5
C6—S2—C5	88.80 (13)	N2—C10—C5	175.0 (4)
C16—S3—C11	88.88 (14)	C12—C11—C17	125.3 (3)
C16—S4—C15	88.73 (13)	C12—C11—S3	115.1 (2)
C2—C1—C7	126.0 (3)	C17—C11—S3	119.6 (2)
C2—C1—S1	114.5 (2)	C11—C12—C13	110.0 (3)
C7—C1—S1	119.5 (2)	C11—C12—C18	125.1 (3)
C1—C2—C3	110.7 (2)	C13—C12—C18	124.9 (3)
C1—C2—C8	124.6 (3)	C16—C13—C14	111.9 (2)
C3—C2—C8	124.6 (2)	C16—C13—C12	112.0 (2)
C6—C3—C4	111.9 (2)	C14—C13—C12	136.0 (3)
C6—C3—C2	112.3 (2)	C15—C14—C13	110.0 (2)
C4—C3—C2	135.9 (2)	C15—C14—C19	123.4 (3)
C5—C4—C3	110.8 (2)	C13—C14—C19	126.5 (3)
C5—C4—C9	124.2 (3)	C14—C15—C20	127.2 (3)
C3—C4—C9	125.0 (2)	C14—C15—S4	114.8 (2)
C4—C5—C10	123.0 (3)	C20—C15—S4	118.0 (2)
C4—C5—S2	114.7 (2)	C13—C16—S4	114.5 (2)
C10—C5—S2	122.2 (2)	C13—C16—S3	114.0 (2)
C3—C6—S1	113.3 (2)	S4—C16—S3	131.48 (17)
C3—C6—S2	113.8 (2)	N3—C17—C11	179.2 (4)
S1—C6—S2	132.84 (17)	C12—C18—H18A	109.5
N1—C7—C1	178.6 (4)	C12—C18—H18B	109.5
C2—C8—H8A	109.5	H18A—C18—H18B	109.5
C2—C8—H8B	109.5	C12—C18—H18C	109.5
H8A—C8—H8B	109.5	H18A—C18—H18C	109.5
C2—C8—H8C	109.5	H18B—C18—H18C	109.5
H8A—C8—H8C	109.5	C14—C19—H19A	109.5
H8B—C8—H8C	109.5	C14—C19—H19B	109.5
C4—C9—H9A	109.5	H19A—C19—H19B	109.5
C4—C9—H9B	109.5	C14—C19—H19C	109.5
H9A—C9—H9B	109.5	H19A—C19—H19C	109.5
C4—C9—H9C	109.5	H19B—C19—H19C	109.5
H9A—C9—H9C	109.5	N4—C20—C15	177.4 (4)
			
C6—S1—C1—C2	0.4 (2)	C16—S3—C11—C12	0.6 (2)
C6—S1—C1—C7	−178.9 (2)	C16—S3—C11—C17	180.0 (2)
C7—C1—C2—C3	179.1 (3)	C17—C11—C12—C13	−179.8 (3)
S1—C1—C2—C3	−0.1 (3)	S3—C11—C12—C13	−0.4 (3)
C7—C1—C2—C8	0.5 (5)	C17—C11—C12—C18	0.3 (5)
S1—C1—C2—C8	−178.6 (2)	S3—C11—C12—C18	179.7 (2)
C1—C2—C3—C6	−0.3 (3)	C11—C12—C13—C16	0.0 (3)
C8—C2—C3—C6	178.3 (2)	C18—C12—C13—C16	179.8 (2)
C1—C2—C3—C4	−179.5 (3)	C11—C12—C13—C14	178.1 (3)
C8—C2—C3—C4	−1.0 (5)	C18—C12—C13—C14	−2.0 (5)
C6—C3—C4—C5	−0.6 (3)	C16—C13—C14—C15	0.6 (3)
C2—C3—C4—C5	178.7 (3)	C12—C13—C14—C15	−177.5 (3)
C6—C3—C4—C9	179.9 (2)	C16—C13—C14—C19	−178.2 (2)
C2—C3—C4—C9	−0.9 (5)	C12—C13—C14—C19	3.7 (5)
C3—C4—C5—C10	−176.6 (3)	C13—C14—C15—C20	178.6 (3)
C9—C4—C5—C10	3.0 (4)	C19—C14—C15—C20	−2.5 (5)
C3—C4—C5—S2	0.9 (3)	C13—C14—C15—S4	−0.4 (3)
C9—C4—C5—S2	−179.5 (2)	C19—C14—C15—S4	178.5 (2)
C6—S2—C5—C4	−0.8 (2)	C16—S4—C15—C14	0.1 (2)
C6—S2—C5—C10	176.8 (3)	C16—S4—C15—C20	−179.0 (2)
C4—C3—C6—S1	180.00 (18)	C14—C13—C16—S4	−0.6 (3)
C2—C3—C6—S1	0.5 (3)	C12—C13—C16—S4	177.97 (17)
C4—C3—C6—S2	0.0 (3)	C14—C13—C16—S3	−178.12 (18)
C2—C3—C6—S2	−179.51 (18)	C12—C13—C16—S3	0.5 (3)
C1—S1—C6—C3	−0.5 (2)	C15—S4—C16—C13	0.3 (2)
C1—S1—C6—S2	179.6 (2)	C15—S4—C16—S3	177.3 (2)
C5—S2—C6—C3	0.5 (2)	C11—S3—C16—C13	−0.6 (2)
C5—S2—C6—S1	−179.6 (2)	C11—S3—C16—S4	−177.5 (2)
